# Epidemiology of Prostate Cancer in the Republic of Kazakhstan

**Published:** 2019-12

**Authors:** Dariga SMAILOVA, Erlan OSPANOV, Meruert GAZALIYEVA, Dilyara KAIDAROVA, Oxana SHATKOVSKAYA, Zhanar ZAMANBEKOVA, Kuralay AMRENOVA, Tatyana BELIKHINA, Tasbolat ADYLKHANOV, Ardak OMARBEKOV, Marzhan DAULETYAROVA, Lyudmila PIVINA, Yuliya SEMENOVA

**Affiliations:** 1. Department of Innovations in Science, Semey Medical University, Semey, Republic of Kazakhstan; 2. Department of Neurology, Ophthalmology, and ENT, Semey Medical University, Semey, Republic of Kazakhstan; 3. Department of Clinical Immunology, Allergology, and Microbiology, Karaganda Medical University, Karaganda, Republic of Kazakhstan; 4. Kazakh Institute of Oncology and Radiology, Almaty, Republic of Kazakhstan; 5. Department of Endocrinology, Semey Medical University, Semey, Republic of Kazakhstan; 6. Department of Personalized Medicine, Semey Medical University, Semey, Republic of Kazakhstan; 7. Semey Regional Oncology Center, Semey, Republic of Kazakhstan; 8. Department of Oncology, Semey Medical University, Semey, Republic of Kazakhstan; 9. Department of Surgical Diseases ?2, Astana Medical University, Astana, Republic of Kazakhstan; 10. Research Office, Astana Medical University, Astana, Republic of Kazakhstan; 11. Department of Emergency Medicine, Semey Medical University, Semey, Republic of Kazakhstan

**Keywords:** Prostate cancer, Morbidity, Mortality, Screening program, Kazakhstan

## Abstract

**Background:**

The incidence and mortality from prostate cancer in most native Asian populations remain low although a gradual increase is observed over the last years.

**Methods:**

The statistical analysis of official data on prostate cancer mortality and morbidity was performed for the whole country and for Pavlodar Region.

**Results:**

The increase in the incidence of prostate cancer among the population of Kazakhstan is observed, which may be attributed to the introduction of screening program based on serum PSA. Still, the crude incidence rates in Kazakhstan are below world indices. Over the last few years, the decreasing prostate cancer mortality is observed that might be influenced by early diagnosis. The age-standardized incidence rates show that the majority of prostate cancer cases occur in advanced ages (70 years and older).

**Conclusion:**

More research is needed to determine the risk factors for prostate cancer, as well as ethnic and geographical trends for the population of Kazakhstan

## Introduction

Malignant neoplasms rank the second position among the causes of death from noncommunicable diseases, killing 8.2 million people every year (1). In recent decades, there has been a trend towards high-tech clinical research aimed at developing new methods for diagnosis and treatment of malignant tumors. At the same time, studies devoted to the epidemiology of the particular types of cancer were less frequent. Despite this, without understanding the patterns of oncology morbidity, it is impossible to build a quality program aimed at controlling cancer incidence and mortality at the population level. Moreover, development of such programs is strongly associated with patients’ satisfaction (2, 3).

Worldwide, prostate cancer is the second (after lung cancer) commonest malignancy in men (4). In general, prostate cancer is less common in the Asian population as compared to the European, where it is the most common type of cancer in the male population (5). The reasons for this are not only racial characteristics, but also the population aging, which is characteristic of the countries of Western Europe (6). Thus, in 2012, 60% of all new cancer cases and 41% of prostate cancer deaths were noted in Europe and North America (7). At the same time, the highest mortality from prostate cancer is observed in the Caribbean region, where the African population prevails (8). There are more than 25-fold differences in the incidence of prostate cancer among different countries and regions of the world. The highest values are observed in Australia and New Zealand, where the prevalence is as high as 111.6 per 100,000 population (4). In Eastern European states – the former neighbors of Kazakhstan by Soviet block – the significance of prostate cancer as a public health problem is high. Such, in Estonia the age-adjusted incidence rate is 111.4 per 100,000 population (5), the mortality from prostate cancer in Poland is increasing, like in Slovakia and Slovenia (6).

Globally, the increase in the incidence of prostate cancer began in the late 1980s, which was associated with the widespread use of prostate-specific antigen (PSA) as the method of early diagnosis. However, mortality rates from prostate cancer began to decline in the 1990s due to the emergence of new effective treatments and early diagnosis (9).

The aim of this study was to analyze the patterns of morbidity and mortality from prostate cancer in the Republic of Kazakhstan in comparison with Pavlodar region over a period of 10 years (from 2007 to 2016).

## Materials and Methods

To achieve this aim, we conducted an analysis of official statistics on cases of prostate cancer (annual reports of cancer dispensaries of the Republic of Kazakhstan for the newly diagnosed cases of prostate cancer over a period of 10 years (2007–2016)). We also obtained information from the Committee on Statistics of the Ministry of National Economy of the Republic of Kazakhstan on the total number of population, including the number of male population. We estimated morbidity and mortality from prostate cancer per 100,000 of population based on the number of male population. The study design was ecological.

The direct method of standardization was used to calculate the age-standardized (ASR) incidence rate with the following formula:

$$\begin{array}{l} \text{ASR}=\sum_{j=1}^{j}wj\frac{aj}{n}\\ \mathrm{SE(ASR)}=\sqrt{(\sum_{j=1}^{j}wj\frac{aj}{n}}/n^{2}j\\ 95\%\text{CI}=\text{ASR}\pm 1.96*\text{SE(ASR)} \end{array}$$

where aj is a number of disease cases in a specific age group of a study population at a period of time; nj is a number of person-years in a given age group of the same population over the studied time period, wj is a number of population in a given age group of a standard population, SE is a standard error and CI is a confidence interval.

The statistical analysis was carried out using the SPSS statistical package (Statistical Package for the Social Sciences) version 20.0 for Windows (license of the GMU of Semey). Before our study was started, we received the approval of the Ethics Committee of the Semey State Medical University.

## Results

There did not appear to be significant variation in crude incidence rates of major cancer types in the Republic of Kazakhstan over 2007–2016. In this period of time, breast, lung, stomach, cervix uteri and esophagus were the most predominant cancer sites. Despite this, there was a shift in the incidence of prostate cancer, which increased from 8.5% in 2007 to 17.8% in 2016 (Table 1).

**Table 1:** Crude incidence rates of major cancer types, both sexes, in the Republic of Kazakhstan over the period of 2007–2016 (per 100,000 population)

***Cancer sites***	***Years***										
	***2007***	***2008***	***2009***	***2010***	***2011***	***2012***	***2013***	***2014***	***2015***	***2016***
Trachea, bronchi and lung	22.6	23	22.4	21.7	20.9	21.8	22	20.2	21.5	19.4
Stomach	18.8	17.4	16.9	16.3	16.2	16.3	16.4	15.7	16.0	14.9
Breast	37.4	38.6	38.2	38.6	39.9	44.2	42.4	45.5	47.5	49.0
Esophagus	9.1	8.2	8.2	8.1	8.1	8.1	7.3	7.4	7.5	7.0
Colon	7.6	7.4	7.7	8.5	8	8.7	9	8.8	9.1	9.0
Rectum	7.4	7.7	7.2	7.2	7.5	7.8	8.3	8.1	7.8	7.9
Cervix uteri	14.9	14.9	15.8	15.7	16.5	18.2	18.0	19.7	19.8	18.3
Prostate	8.5	7.5	8.0	8.5	10.2	10.8	13.2	15.1	15.8	17.8

To compare the incidence of malignant neoplasms with world indices, we extracted the data from World Cancer Report for 2012 (1) and linked them to the data from Kazakhstan for the same year (Table 2).

**Table 2:** Incidence of top 10 malignant neoplasms, both sexes, Kazakhstan and worldwide in 2012 (per 100,000 population)

***Cancer sites***	***Worldwide***	***Kazakhstan***
Breast	47.8	44.2
Prostate	30.8	10.8
Lung	25.9	21.0
Colorectum	19.3	13.2
Cervix uteri	15.1	18.2
Stomach	13.5	16.3
Liver	11.1	4.5
Corpus uteri	9.1	11.1
Ovary	6.8	11.3
Esophagus	6.5	8.1
All cancers excluding non-melanoma skin	199.4	178.2

**Fig. 1:**
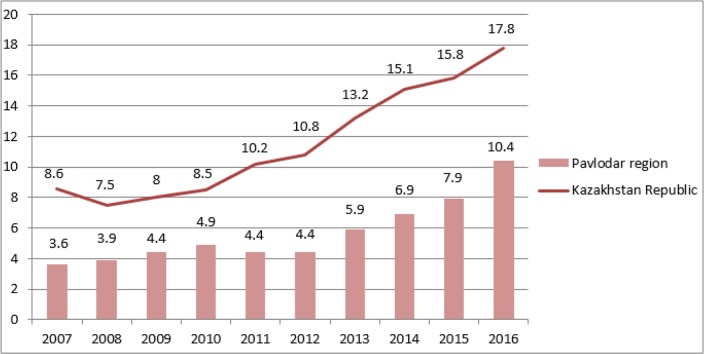
Standardized incidence rate of prostate cancer in the Kazakhstan Republic and Pavlodar region per 100,000 male population

**Fig. 2:**
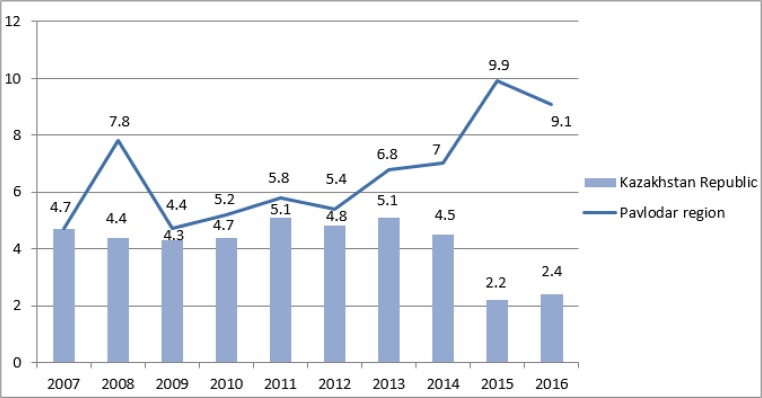
Standardized mortality rate from prostate cancer in the Kazakhstan Republic and Pavlodar region per 100,000 male population

The standardized morbidity and mortality rates for 2007–2016 from to prostate cancer in the Republic of Kazakhstan in comparison with the Pavlodar region are presented in Figs. 1 and 2.

Over the past two years, there has been a decrease in mortality rates from prostate cancer in the Republic of Kazakhstan, which contradicts the growth of mortality from this disease in the Pavlodar region, which reached its peak in 2015. However, beginning 2016 a trend for mortality decrease was observed. In general, the standardized mortality rate from prostate cancer in Pavlodar region was higher than the average national indices. Nevertheless, over the same time period the regional morbidity rate was below the average national indices.

Data on age distribution of the prostate cancer incidence among male population of the Pavlodar region for a ten-year period (2007–2016) are summarized in Table 3.

**Table 3:** Incidence (Inc) of prostate cancer in the Pavlodar region by age groups for the period from 2007 to 2016

***Age (yr)***	***Years***										
	***2007 N (Inc)***	***2008 N (Inc)***	***2009 N (Inc)***	***2010 N (Inc)***	***2011 N (Inc)***	***2012 N (Inc)***	***2013 N (Inc)***	***2014 N (Inc)***	***2015 N (Inc)***	***2016 N (Inc)***
<29	1 (0.5)	0	0	0	0	0	0	0	0	0
30–39	0	0	0	0	0	0	0	0	0	0
40–49	3 (5.5)	0	0	1 (1.9)	2 (3.8)	0	1 (1.9)	0	1 (1.8)	4 (7.2)
50–59	4 (11.0)	6 (16.0)	7 (18.5)	6 (15.3)	4 (9.8)	4 (9.5)	13 (29.7)	15 (33.3)	11 (23.9)	18 (38.8)
60–69	20 (104.9)	13 (70.9)	15 (80.4)	16 (86.0)	17 (90.7)	19 (99.3)	40 (200.6)	39 (182.9)	39 (169.5)	62 (239.4)
>70	14 (117.5)	30 (234.2)	28 (196.3)	21 (139.8)	19 (122.0)	23 (146.5)	43 (277.8)	39 (263.1)	50 (353.2)	64 (464.9)
Total	42 (11.9)	49 (13.9)	50 (14.3)	44 (12.6)	42 (11.9)	46 (13.1)	97 (27.5)	104 (29.3)	101 (28.3)	148 (41.3)

As Table 3 shows, the peak incidence of prostate cancer falls at the age of 70 years and older, while only a few cases of this disease are noted in males aged less than 40 years of age. Beginning from 2013, the incidence of prostate cancer in Pavlodar region has increased more than twofold, which coincides with the start of prostate cancer’s screening program.

**Fig. 3:**
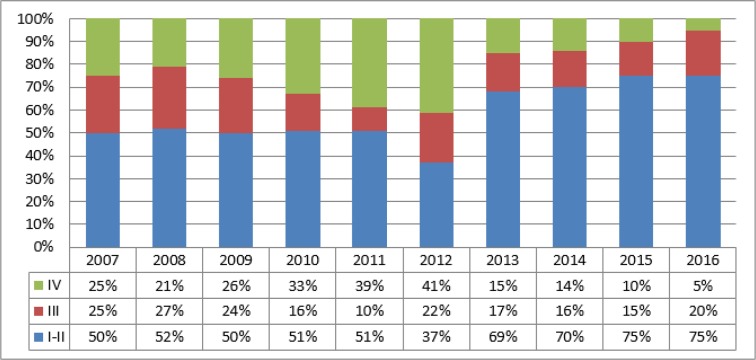
Distribution of prostate cancer stages in Pavlodar region over the period of study (2007 to 2016)

Figure 3 shows the distribution of prostate cancer cases by the disease stages at the time of diagnosis, for a period of 10 years (2007–2016) in Pavlodar region.

As follows from Figure 3, in the period of 2007–2009 the majority of prostate cancer cases observed in Pavlodar region belonged to the 1^st^ and 2^nd^ stages of the disease, except for 2012 when only 37% of patients were identified at these stages. Still, since 2013, there has been almost a twofold increase in the number of cases of prostate cancer identified at first two stages of the disease, which corresponds with initiation of screening program. Basically, introduction of screening for prostate cancer helped to decrease significantly the proportion of cases detected at the 4^th^ stage to as low as 5% by 2016.

## Discussion

The increase in the incidence of prostate cancer in Kazakhstan may be associated with the introduction of screening program that started in 2013 in 5 pilot regions, including Pavlodar region. In 2014, the geography of the program has expanded and it began to cover 5 more regions of the country. In Kazakhstan, screening for prostate cancer is carried out by examining the serum PSA levels (prostate-specific antigen) every 4 years among men aged 50–66 years who are not followed for this disease. The recent study suggests that introduction of prostate cancer screening by means of PSA testing has substantially contributed to the improved diagnostics of this disease since 83% of new cases are being diagnosed at stage I or II (10).

In general, the evidence that prostate cancer screening reduces the mortality from this disease is not fully convincing. Although screening programs are able to detect the early stages of prostate cancer, which contributes to early treatment, it is not completely clear how this will affect the natural course of the disease and its outcomes (11). There are studies that show a trend towards reduction in prostate cancer mortality in a number of countries, but it is not completely clear to what extent this is due to screening and not due to improvements in the treatment of prostate cancer (12). In addition, screenings create a burden on the health care system, provoking a number of problems associated with over-diagnosis of prostate cancer and an increase in the financial costs of screening by itself as well as the treatment of newly diagnosed patients (13–15). Pavlodar region is characterized by higher rates of morbidity and mortality from prostate cancer, which, similar to the neighboring East Kazakhstan region, might be partly explained by industrialization and westernized style of living (16). Still, this region was also affected by the activity of Semipalatinsk Nuclear Test Site, which had many negative impacts on population health apart from cancer disease (17; 18). It has to be noted that a part of the Semipalatinsk Nuclear Test Site is located on the territory of Pavlodar region, but major part is covering East Kazakhstan region (19, 20).

However, the incidence of prostate cancer in the Republic of Kazakhstan is lower than in the industrialized countries of the world, which is generally characteristic of the Asian population and can be associated with both lifestyle and overall life expectancy (21). Such, in China, Korea and Japan the age-standardized incidence rate for prostate cancer is 12.0, 22.4, 26.6 per 100,000 male population, respectively (22). At the same time, in Europe, North America, Australia and New Zealand, prostate cancer is the main oncology disease among the male population and accounts for 22.8% (23), 28.3% and 30.4% (24), respectively.

In the Russian Federation (25) and in the Republic of Belarus (26), prostate cancer holds the second place among all oncology disorders in men. In Saint-Petersburg alone the age-standardized mortality rates from prostate cancer per 100,000 population are being increasing from 15.1 in 2000 to 18.1 in 2005, 21.1 in 2010 and 22.6 in 2011 (27). Generally, Belarus, Russia and Kazakhstan are three member countries of the Commonwealth of Independent States with the highest rates of cancer incidence in males (312.5, 270.8 and 196.8 per tens of thousands, respectively) (28).

In Ukraine the frequency of prostate cancer in men is equal to 8.4%, while the crude age-standardized rate is 27.9 per 100,000 population (5). The prostate cancer incidence rates in Georgia are consistently higher the national average (approximately 167 versus 162 cases per 100,000 male population, 2000–2010), which may be due to agricultural practices, associated with the use of commercial fertilizers – the known risk factor for this disorder (29).

In other countries of the Central Asian region, like in Kazakhstan, the proportion of prostate cancer in the overall structure of cancer morbidity remains low. For instance, in 2012 the incidence of prostate cancer was equal to 3.6 in Uzbekistan and 7.5 in Kyrgyzstan, while the mortality was 1.6 and 3.4, respectively. At the same time, the majority of patients (46.9% in Uzbekistan and 39.2% in Kyrgyzstan) had stage III of the disease at the time of diagnosis (30).

## Conclusion

The study shows an increase in the incidence of prostate cancer among the population of Kazakhstan for the period 2007–2016, which may be due to the introduction of screening program by testing serum PSA. There has been a decrease in mortality from prostate cancer in the Republic of Kazakhstan during the last two years (2015–2016) of this time period. The peak incidence of prostate cancer falls at the age of 70 years and older. Since 2013, there has been an increase in the identification of prostate cancer in the early (1–2) stages in Pavlodar region, which corresponds with initiation of the screening program. More research is needed to determine the risk factors for prostate cancer, as well as ethnic and geographical trends for the population of Kazakhstan.

## Ethical considerations

Ethical issues (Including plagiarism, informed consent, misconduct, data fabrication and/or falsification, double publication and/or submission, redundancy, etc.) have been completely observed by the authors.

## References

[1] WHO (2014). World cancer report. Geneva, 632 pages.

[2] Dauletyarova M Semenova Y Kaylubaeva G (2016). Are Women of East Kazakhstan Satisfied with the Quality of Maternity Care? Implementing the WHO Tool to Assess the Quality of Hospital Services. Iran J Public Health, 45(6): 729–38. 27648415 PMC5026827

[3] Dauletyarova MA Semenova YM Kaylubaeva G (2018). Are Kazakhstani Women Satisfied with Antenatal Care? Implementing the WHO Tool to Assess the Quality of Antenatal Services. Int J Environ Res Public Health, 15:E325. 29438330 10.3390/ijerph15020325 PMC5858394

[4] Ferlay J Bray F Pisani P Parkin DM (2001). GLOBOCAN 2000: Cancer Incidence, Mortality and Prevalence Worldwide. IARC Cancer Base No. 5, Lyon: IARC.

[5] Bray F Ren JS Masuyer E Ferlay J (2013). Global estimates of cancer prevalence for 27 sites in the adult population in 2008. Int J Cancer, 132: 1133–1145. 22752881 10.1002/ijc.27711

[6] Forman D Bray F Brewster DH (2014). Cancer Incidence in Five Continents, Vol. X [electronic version]. Lyon: IARC Available from: http://ci5.iarc.fr

[7] Giovannucci E Platz EA Mucci L (2011). Epidemiology of prostate cancer. In: Comprehensive Textbook of Genitourinary Oncology. Eds, Scardino Linehan Zelefsky 4th ed, Lippincott Williams & Wilkins Baltimore, pp. 1–17.

[8] Levi F Lucchini F Negri E Boyle P La Vecchia C (2004). Leveling of prostate cancer mortality in Western Europe. Prostate, 60(1): 46–52. 15129428 10.1002/pros.20058

[9] Henderson BE Lee NH Seewaldt V Shen H (2012). The influence of race and ethnicity on the biology of cancer. Nat Rev Cancer, 12: 648–653. 22854838 10.1038/nrc3341 PMC3677949

[10] Ospanov E Adylkhanov T Tokanova Sh (2017). Mortality and morbidity from prostate cancer in the Republic of Kazakhstan from 2007 to 2016. Georgian Med News, (272): 17–22. 29227252

[11] Ishkinin YI Zhylkaidarova A Nurgaliyev N (2017). Population-based Prostate Cancer Screening in Kazakhstan. Iran J Public Health, 46(7): 917–922. 28845402 PMC5563873

[12] PDQ Screening and Prevention Editorial Board (2017). Prostate Cancer Screening. National Cancer Institute, Bethesda (USA) Available from: https://www.ncbi.nlm.nih.gov/books/NBK65945/

[13] Moyer VA, U.S (2012). Preventive Services Task Force: Screening for prostate cancer: US Preventive Services Task Force recommendation statement. Ann Intern Med, 157 (2): 120–34. 22801674 10.7326/0003-4819-157-2-201207170-00459

[14] Djulbegovic M Beyth RJ Neuberger MM (2010). Screening for prostate cancer: systematic review and meta-analysis of randomized controlled trials. BMJ, 341: c4543. 20843937 10.1136/bmj.c4543 PMC2939952

[15] Ilic D O’Connor D Green S Wilt T (2007). Screening for prostate cancer: a Cochrane systematic review. Cancer Causes Control, 18: 279–285. 17206534 10.1007/s10552-006-0087-6

[16] Yelissinova N Grjibovski AM Yelissinova A (2015). Sociodemographic factors associated with infant abandonment in maternity hospitals in Kazakhstan: A case-control study. Public health, 129(7): 1010–1013. 25986952 10.1016/j.puhe.2015.04.009

[17] Dyussenova L Pivina L Semenova Y (2018). Associations between depression, anxiety and medication adherence among patients with arterial hypertension: comparison between persons exposed and non-exposed to radiation from the Semipalatinsk Nuclear Test Site. J Environ Radioact, 195: 33–39. 30241015 10.1016/j.jenvrad.2018.09.016

[18] Markabayeva A Bauer S Pivina (2018). Increased prevalence of essential hypertension in areas previously exposed to fallout due to nuclear weapons testing at the Semipalatinsk test site, Kazakhstan. Environ Res, 167: 129–135. 30014894 10.1016/j.envres.2018.07.016

[19] Shalgumbayeva GM Rakhypbekov TK Sagidullina GG (2014). Incidence of and mortality from cervical cancer in the territory adjacent to the former Semipalatinsk nuclear test site in 2008–2012. Ekologiia Cheloveka, 5(1): 41–47.

[20] Schwerin M Schonfeld S Drozdovitch V (2010). The utility of focus group interviews to capture dietary consumption data in the distant past: dairy consumption in Kazakhstan villages 50 years ago. J Dev Orig Health Dis, 1(3): 192–202. 24286002 10.1017/S2040174410000243 PMC3839237

[21] Parkin DM Ferlay J Curado MP (2010). Fifty years of cancer incidence: CI5 I-IX. Int J Cancer, 127: 2918–2927. 21351270 10.1002/ijc.25517

[22] Ito K (2014). Prostate cancer in Asian men. Nat Rev Urol, 11(4): 197–212. 24595118 10.1038/nrurol.2014.42

[23] Ferlay J Shin HR Bray F (2010). Estimates of worldwide burden of cancer in 2008: GLOBOCAN 2008. Int J Cancer, 127: 2893–2917. 21351269 10.1002/ijc.25516

[24] Center MM Jemal A Lortet-Tieulent J (2012). International variation in prostate cancer incidence and mortality rates. Eur Urol, 61: 1079–92. 22424666 10.1016/j.eururo.2012.02.054

[25] Merabishvili VM Petrova NG Atroshchenko AV Kharitonov MV (2014). [Epidemiology of prostate cancer (cohort study)]. Vopr Onkol, 60(4): 457–63. 25552064

[26] Zalutskii˘ IV Zhavrid EA Mashevskii˘ AA (2009). [Oncological service in the Republic of Belarus in 2008 and prospects for developmen]t. Vopr Onkol, 55(5): 555–61. 20020649

[27] Merabishvili VM (2017). [Age-related risks of cancer (analytical indicators of registration and early diagnosis)]. Adv Gerontol, 30(6): 818–825. 29608822

[28] Davydov MI Aksel' EM (2007). The incidence of malignant tumors and mortality caused by them in Commonwealth of Independent States in 2005. Vestn Ross Akad Med Nauk, (11): 45–9. 18080527

[29] Welton M Robb SW Shen Y Guillebeau P Vena J (2015). Prostate cancer incidence and agriculture practices in Georgia, 2000–2010. Int J Occup Environ Health, 21(3): 251–7. 25785490 10.1179/2049396714Y.0000000106 PMC4597014

[30] Golovachev SV Nurgaliev NS Kamarli ZP Makimbetov EK (2016). Status of oncological care and epidemiology of prostate cancer in the republics of Central Asia. Cancer Urology, 12(3): 82–86. [Article in Russian]

